# Unicentric Hyaline-Vascular Castleman Disease Presenting as a Periduodenal Mass: A Rare Retroperitoneal Manifestation

**DOI:** 10.7759/cureus.90156

**Published:** 2025-08-15

**Authors:** Susan Feldman, Alekhya Mitta, Laine Lyles, Devendra Enjamuri, Kenneth J Vega

**Affiliations:** 1 Internal Medicine, Prisma Health Midlands, Columbia, USA; 2 Medicine, University of South Carolina School of Medicine Columbia, Columbia, USA; 3 Gastroenterology and Hepatology, Prisma Health Midlands, Columbia, USA

**Keywords:** castleman disease, endoscopic ultrasound, oncology, retroperitoneal, surgery

## Abstract

Castleman disease (CD) is a rare lymphoproliferative disorder, typically presenting in the mediastinum. We report a case of a 46-year-old male with back and abdominal pain found to have an incidental 3.9 cm retroperitoneal mass posterior to the duodenum. Endoscopic biopsy revealed atypical B-cell proliferation, and surgical resection confirmed unicentric hyaline-vascular CD. This case highlights a rare periduodenal presentation and underscores the importance of including CD in the differential diagnosis of retroperitoneal masses, even in atypical locations, to ensure timely recognition and management.

## Introduction

Castleman disease (CD), also known as angiofollicular lymph node hyperplasia, is a rare group of lymphoproliferative disorders with a largely unknown etiology [1]. It is classified clinically into unicentric, oligocentric, and multicentric subtypes depending on the number of lymphatic regions involved [1,2]. The unicentric and oligocentric forms typically affect a single or localized group of lymph nodes, are often asymptomatic, and are curable with complete surgical resection. By contrast, multicentric CD is a systemic and progressive form associated with constitutional symptoms, such as fever, night sweats, hepatosplenomegaly, weight loss, and elevated inflammatory markers, features that often mimic those of lymphoma or autoimmune disorders [2,3,4].

Histologically, CD is divided into three types: the hyaline-vascular type (80-90% of cases), the plasma cell type (10-20%), and a mixed variant [2]. The hyaline-vascular subtype, which predominates in unicentric cases, is often discovered incidentally during imaging or surgery. While CD can involve lymph nodes in virtually any region of the body, over 70% of cases are in the mediastinum [3]. Less common sites, such as the abdominal, pelvic, and cervical regions, are more often associated with multicentric disease [1].

We present a case of unicentric, hyaline-vascular CD presenting as a periduodenal mass, a rare retroperitoneal manifestation with limited documentation in the literature. This case contributes to the evolving understanding of CD’s anatomical diversity and highlights the importance of including CD in the differential diagnosis of retroperitoneal soft tissue masses.

## Case presentation

A 46-year-old male with a medical history of type 2 diabetes mellitus presented to his primary care provider with a two-month history of intermittent, dull lower back pain, vague epigastric abdominal discomfort, and new-onset urinary hesitancy. There were no exacerbating or alleviating factors reported by the patient. He denied fever, night sweats, weight loss, changes in bowel habits, or hematuria and had no history of malignancy or recent infections. Physical exam did not reveal lymphadenopathy, cardiac and pulmonary auscultation were normal, the abdomen was notable for periumbilical tenderness to palpation without hepatosplenomegaly, and there was no lower extremity edema.

**Figure 1:**
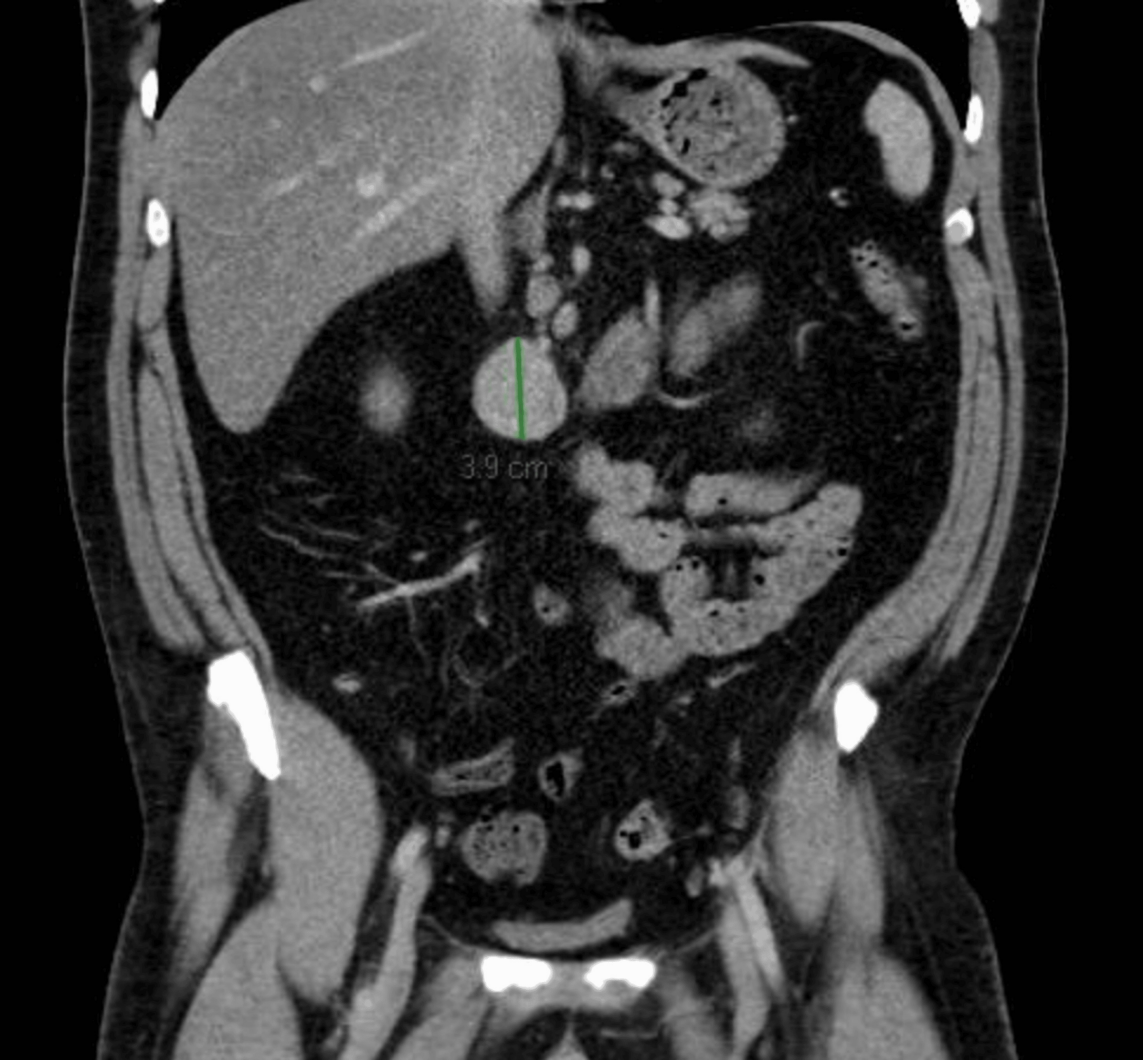
CT abdomen pelvis with IV contrast. Coronal view. The image shows a 3.9 cm craniocaudal measurement.

**Figure 2:**
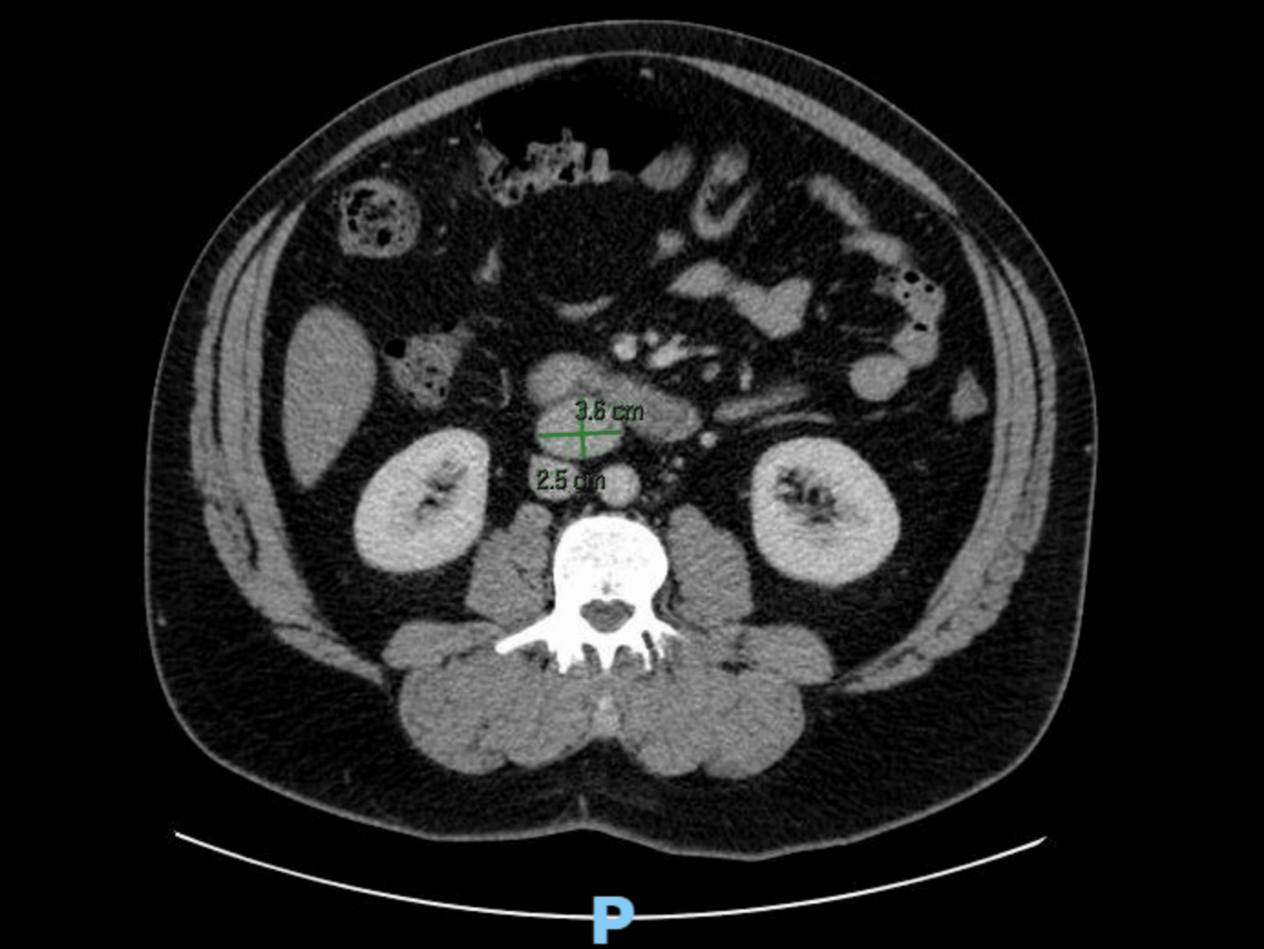
CT abdomen pelvis with IV contrast. Axial view. The image shows a 3.6 cm measurement transverse and 2.5 cm anteroposterior.

Initial clinical suspicion was nephrolithiasis. A CT scan of the abdomen and pelvis with IV contrast was obtained, revealing no renal calculi but incidentally identifying a well-defined 3.6 × 2.5 x 3.9 cm soft tissue retroperitoneal mass posterior to the third portion of the duodenum (Figures 1, 2).

The lesion was not contiguous with bowel, pancreas, kidneys, or major vessels, and there was no evidence of lymphadenopathy elsewhere.

**Figure 3:**
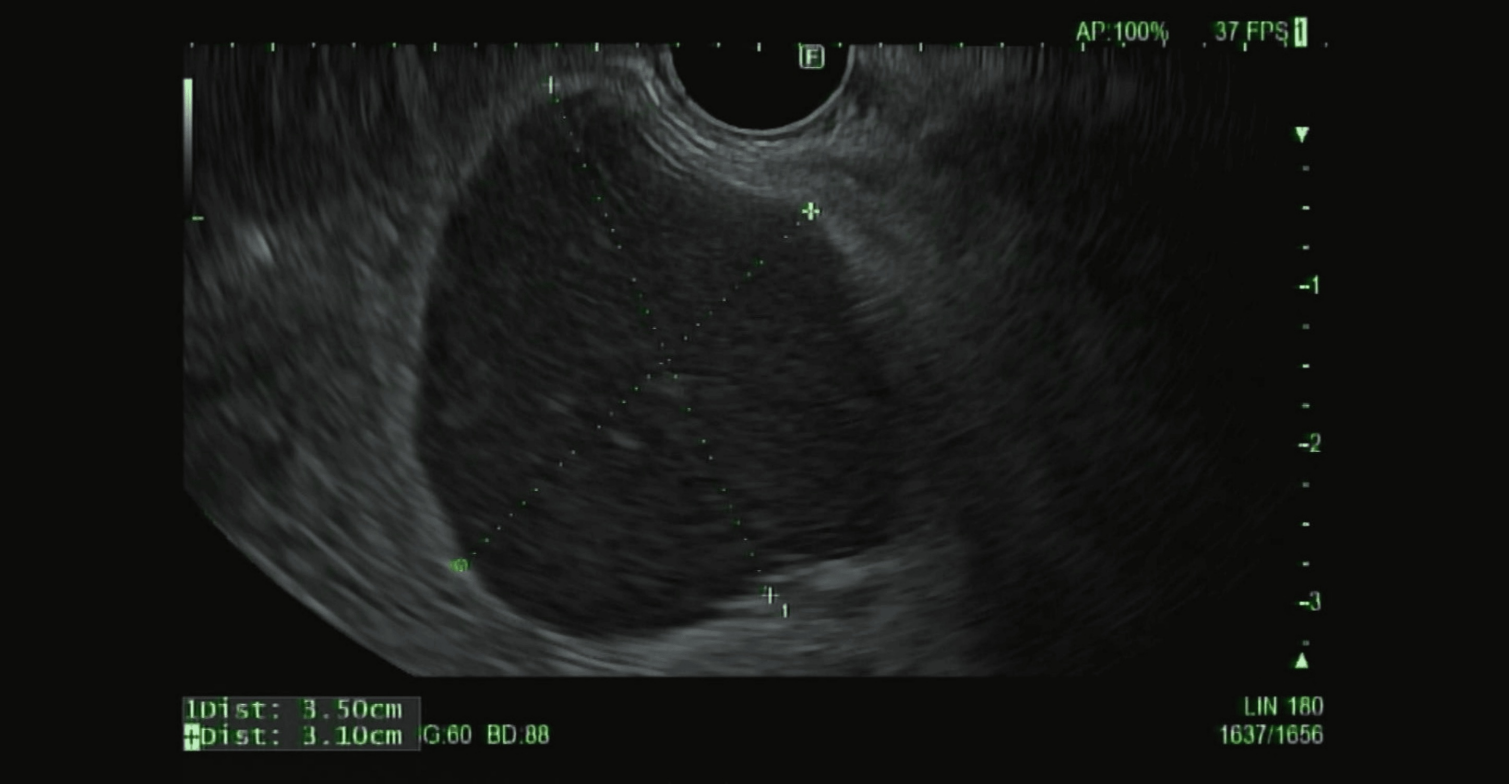
Endoscopic ultrasound image of the mass with dimensions

The patient was referred to gastroenterology for further evaluation. An endoscopic ultrasound (EUS) demonstrated a hypoechoic, well-circumscribed mass with internal vascularity (Figure 3).

EUS-guided fine-needle aspiration revealed atypical B-cell proliferation, although flow cytometry was inconclusive. The clinical differential included gastrointestinal stromal tumor (GIST), lymphoma, or a neuroendocrine neoplasm. Given the indeterminate findings and potential for malignancy, the patient was referred to surgical oncology. He underwent robotic-assisted laparoscopic resection of the retroperitoneal mass. Intraoperatively, the lesion was easily dissected from surrounding tissues and removed en bloc without complication.

**Figure 4:**
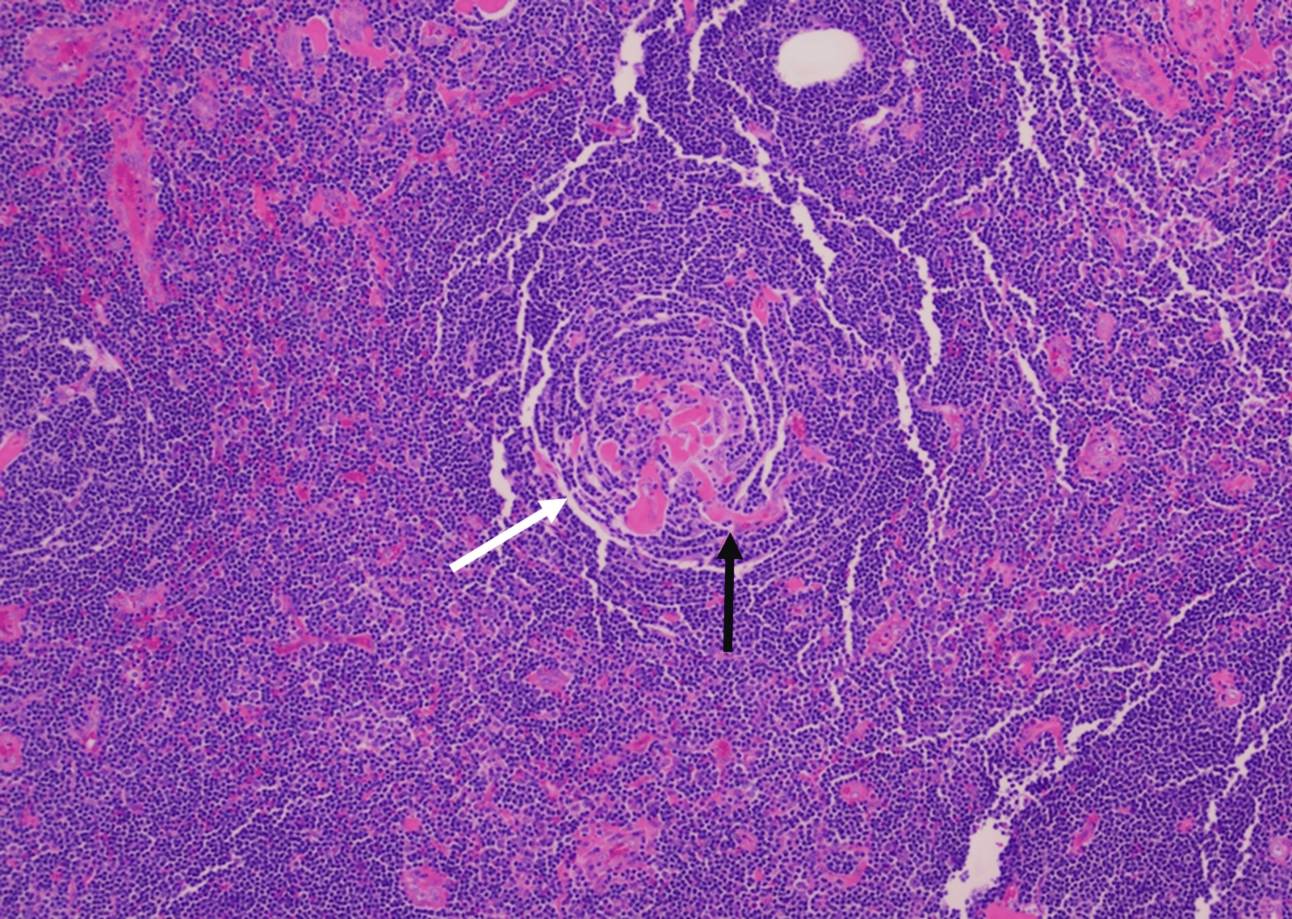
Histology of biopsy showing "onion-skin" appearance (white arrow) and hyalinized vessels penetrating the germinal centers create the classic “lollipop” appearance (black arrow).

**Figure 5:**
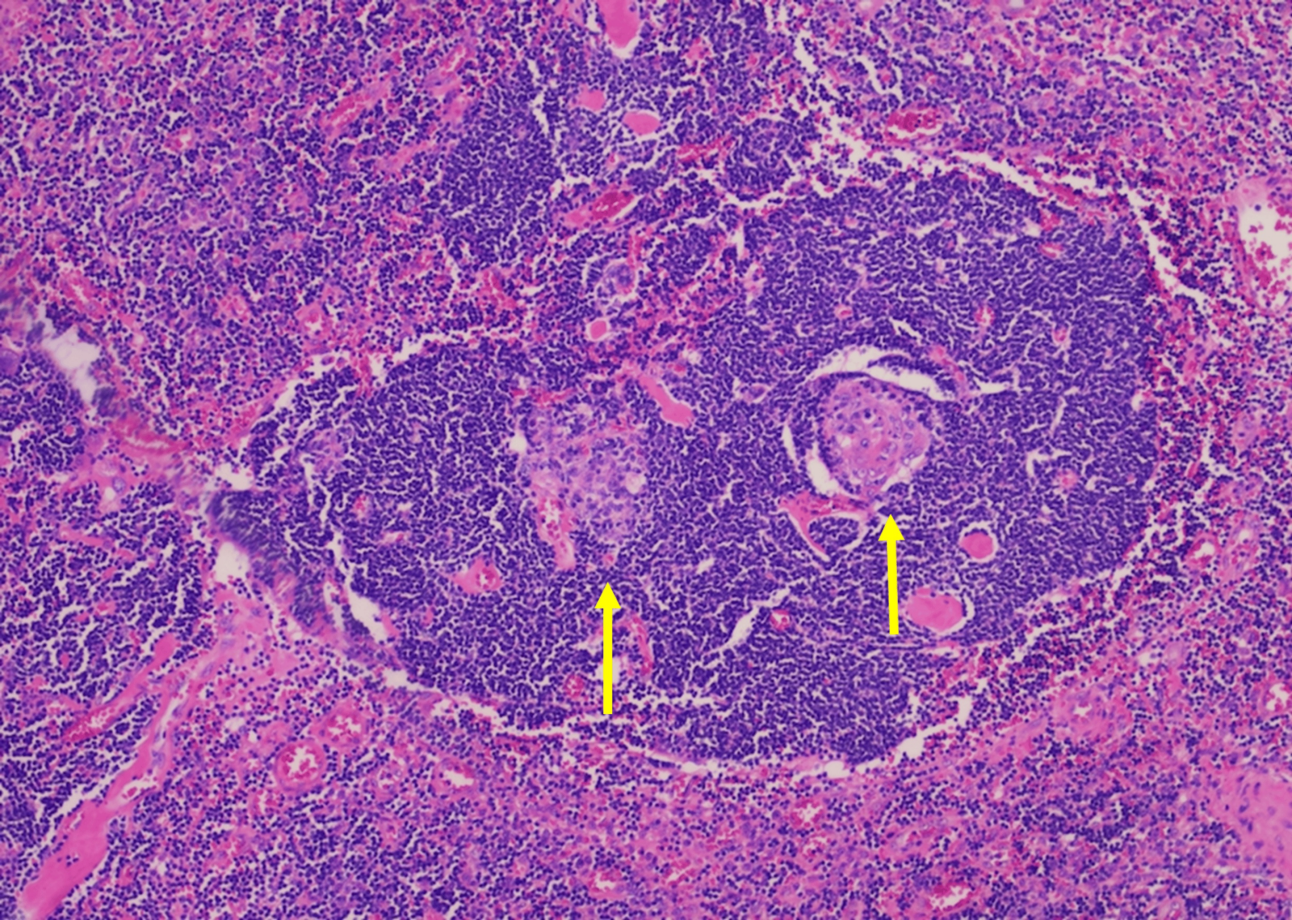
Histology of biopsy demonstrating "twinning" of follicles (yellow arrows).

Gross pathology showed a tan, firm, encapsulated lymphoid mass measuring 4.2 cm. Histologic analysis revealed characteristic features of hyaline-vascular CD, including small, hyalinized follicles with “lollipop” vascular proliferation, concentric layering of mantle zone lymphocytes (“onion-skinning”), and occasional "twinning" of follicles-fused germinal centers sharing an expanded mantle zone (Figures 4, 5).

The patient’s postoperative course was uneventful, with discharge on postoperative day two. At six-month surgical oncology follow-up, he remained asymptomatic, except for mild periumbilical tenderness to pressure, with no evidence of recurrence on surveillance imaging.

## Discussion

CD is a rare, often misunderstood lymphoproliferative disorder with a spectrum of clinical behavior ranging from indolent and localized to aggressive and systemic. The unicentric hyaline-vascular variant, as observed in our patient, is typically asymptomatic and found incidentally during imaging for unrelated complaints [1,5]. The periduodenal retroperitoneum is an uncommon location for CD. While the mediastinum is the most frequently involved site (seen in up to 70% of cases), extrathoracic locations, such as the retroperitoneum, pelvis, or neck, account for a minority of cases [6]. This case, therefore, represents a particularly unusual anatomic presentation of unicentric CD, contributing to the limited number of similar reports in the literature [7,8].

The diagnostic workup of retroperitoneal masses can be challenging. Cross-sectional imaging may reveal a vascular, well-circumscribed lesion, but it is rarely definitive, as a gastrointestinal stromal tumor or lymphoma may have a similar appearance. EUS-guided biopsy, while minimally invasive, may be limited in providing adequate tissue for definitive histopathologic evaluation, as was the case here. Surgical excision not only confirms the diagnosis but is also curative in unicentric CD [2,6].

Importantly, multicentric CD is often associated with human herpesvirus-8 (HHV-8), especially in individuals with HIV. In HHV-8-positive multicentric CD, viral infection drives overproduction of interleukin-6, leading to widespread lymphadenopathy, inflammatory symptoms, and an increased risk of progression to lymphoma. These patients often present with constitutional symptoms and laboratory abnormalities such as anemia and elevated inflammatory markers [3]. By contrast, unicentric CD, particularly the hyaline-vascular subtype, is not associated with HHV-8 or HIV and generally occurs in immunocompetent individuals without systemic symptoms, features consistent with our patient. Histologically, the hyaline-vascular type of CD is characterized by regressed germinal centers, increased vascularity, and proliferation of follicular dendritic cells. The plasma cell variant, by contrast, features sheets of mature plasma cells and is more likely to present with systemic symptoms, particularly in the setting of multicentric disease [3,5].

The management of CD depends on the subtype. For unicentric disease, complete surgical resection offers excellent outcomes with cure rates exceeding 90% [1,6]. Recurrence is rare but may occur with incomplete excision or in cases with atypical histologic features. By contrast, multicentric CD often requires systemic therapy, including corticosteroids, chemotherapy, immunomodulators, or anti-IL-6 monoclonal antibodies (e.g., siltuximab) [2,4].

This case serves as a reminder that CD should remain in the differential for well-circumscribed retroperitoneal masses, even in the absence of systemic symptoms or typical mediastinal location. It also illustrates the importance of histopathologic confirmation and the utility of robotic resection in achieving diagnostic clarity with minimal morbidity.

## Conclusions

We present a rare case of unicentric hyaline-vascular CD presenting as a periduodenal retroperitoneal mass. This case underscores the diagnostic challenges associated with CD in atypical locations and emphasizes the importance of considering it in the differential diagnosis of retroperitoneal lesions. Surgical excision remains the mainstay of treatment for unicentric disease and offers curative outcomes in most cases. Greater awareness of this rare condition can facilitate timely diagnosis and appropriate management.
